# Speech perception performance of subjects with type I diabetes mellitus in noise^☆^

**DOI:** 10.1016/j.bjorl.2016.07.003

**Published:** 2016-08-09

**Authors:** Bárbara Cristiane Sordi Silva, Erika Barioni Mantello, Maria Cristina Foss Freitas, Milton César Foss, Myriam de Lima Isaac, Adriana Ribeiro Tavares Anastasio

**Affiliations:** a Universidade de São Paulo (USP), Faculdade de Medicina de Ribeirão Preto (FMRP), Departamento de Oftalmologia, Otorrinolaringologia e Cirurgia de Cabeça e Pescoço, Ribeirão Preto, SP, Brazil; b Universidade de São Paulo (USP), Faculdade de Medicina de Ribeirão Preto (FMRP), Divisões de Endocrinologia e Metabologia e Nutrição, Ribeirão Preto, SP, Brazil

**Keywords:** Type 1 diabetes mellitus, Speech perception, Hearing, Auditory perception, *Diabetes mellitus* tipo I, Percepção de fala, Audição, Percepção auditiva

## Abstract

**Introduction:**

Diabetes mellitus (DM) is a chronic metabolic disorder of various origins that occurs when the pancreas fails to produce insulin in sufficient quantities or when the organism fails to respond to this hormone in an efficient manner.

**Objective:**

To evaluate the speech recognition in subjects with type I diabetes mellitus (DMI) in quiet and in competitive noise.

**Methods:**

It was a descriptive, observational and cross-section study. We included 40 participants of both genders aged 18–30 years, divided into a control group (CG) of 20 healthy subjects with no complaints or auditory changes, paired for age and gender with the study group, consisting of 20 subjects with a diagnosis of DMI. First, we applied basic audiological evaluations (pure tone audiometry, speech audiometry and immittance audiometry) for all subjects; after these evaluations, we applied Sentence Recognition Threshold in Quiet (SRTQ) and Sentence Recognition Threshold in Noise (SRTN) in free field, using the List of Sentences in Portuguese test.

**Results:**

All subjects showed normal bilateral pure tone threshold, compatible speech audiometry and “A” tympanometry curve. Group comparison revealed a statistically significant difference for SRTQ (*p* = 0.0001), SRTN (*p* < 0.0001) and the signal-to-noise ratio (*p* < 0.0001).

**Conclusion:**

The performance of DMI subjects in SRTQ and SRTN was worse compared to the subjects without diabetes.

## Resumo

**Introdução:**

O *diabetes mellitus*(DM) e um disturbio metabolico cronico de varias origens, que ocorre quando o pancreas deixa de produzir insulina em quantidade suficiente ou quando o organismo nao consegue responder a esse hormonio de maneira eficiente.

**Objetivo:**

Avaliar o reconhecimento de fala em individuos com *diabetes mellitus* tipo I (DMI) no silencio e no ruido competitivo.

**Método:**

Estudo descritivo, observacional e transversal. Foram incluidos 40 participantes de ambos os sexos entre 18 e 30 anos, divididos em um grupo controle (GC) de 20 individuos saudaveis sem queixas ou alteraçoes auditivas, pareados por idade e sexo com o grupo de estudo, composto por 20 individuos com diagnostico de DMI. Inicialmente aplicou-se uma avaliaçao audiologica (audiometria tonal, logoaudiometria e imitanciometria) para todos os individuos; a seguir, os mesmos foram avaliados para o Limiar de Reconhecimento de Sentenças no Silencio (LRSS) e Limiar de Reconhecimento de Sentenças no Ruido (LRSR), em campo livre, por meio do teste Lista de Sentenças em Portugues.

**Resultados:**

Todos os participantes apresentaram audiometria tonal dentro dos padroes de normalidade bilateralmente, logoaudiometria compativel e curva timpanometrica do tipo A. A comparaçao dos grupos revelou uma diferença estatisticamente significante para LRSS (*p* = 0,0001), LRSR (*p* < 0,0001) e a relaçao sinal-ruido (*p* < 0,0001).

**Conclusões:**

O desempenho dos individuos com DMI para LRSS e LRSR foi pior em comparaçao com os individuos sem diabetes.

## Introduction

Diabetes mellitus (DM) is a chronic metabolic disorder of various origins that begins when the pancreas fails to produce insulin in sufficient quantities or when the organism fails to respond to this hormone in an efficient manner. This leads to a condition characterized by hyperglycemia, which may damage certain organs, especially the eyes, kidneys, nerves, heart, and blood vessels.^1^, ^2^

One of the less explored or reported consequences for patients with diabetes is dysfunction of the auditory system.^3^ Patients with DMI do not secrete endogenous insulin or do so in a reduced manner due to the destruction of their pancreatic beta-cells. This situation affects about 20% of cases and is more commonly identified in children and adolescents.^2^ In Brazil there are no studies reporting the exact prevalence of chronic complications in diabetic patients, such as retinopathy, nephropathy, neuropathy, arterial hypertension, cardiovascular changes, and otoneurologic symptoms such as tinnitus, vertigo and hearing loss.^4^, ^5^, ^6^ This may be due to two particular factors: affected persons being unaware of their diseases, and the fact that, although aware of their chronic metabolic disorders, several affected individuals do not seek for medical care.^4^

Some studies have aimed to determine the relationship between DM and hearing loss, but there is no consensus in the international literature about the correlation between these two conditions. No definite cause–effect relationship has been confirmed between diabetes and deafness, and controversies regarding the audiological and histopathological findings attributed to diabetes still remain.^2^, ^3^, ^7^, ^8^, ^9^

Changes in the hearing function of diabetic patients have been identified through audiometry evaluations,^7^, ^8^, ^9^, ^10^ through otoacoustic emissions,^11^, ^12^, ^13^, ^14^ and through brainstem auditory evoked potentials.^8^, ^12^, ^13^, ^15^, ^16^, ^17^ Several studies have investigated the mechanism by which changes in glucose and insulin may produce changes in hearing perception and vestibular function. Labyrinthine structures, especially the stria vascularis, are known to be extremely metabolically active, and thus are susceptible to oxygen and glucose levels and to the availability of adenosine triphosphate, necessary for the preservation of the endocochlear potential. Thus, glucose metabolism significantly influences the normal performance of the inner ear considering that both hypoglycemia and hyperglycemia could impair its average performance.^18^, ^19^

Today there is no consensus in the international literature concerning the etiopathogenesis of hearing loss in diabetics. Some investigators have argued that hearing changes may occur because of neuropathy while others have claimed they occur due to angiopathy. Some investigations also combine the two causes.^7^, ^8^, ^9^, ^10^, ^11^, ^12^, ^13^, ^15^, ^16^, ^17^, ^18^, ^19^

The peripheral and central integrity of the auditory system is essential for an appropriate speech perception. Most sensorineural hearing losses initially affect ultra-high frequencies, that are necessary for consonant discrimination and speech recognition. Impairment of speech perception occurs because although vowel sounds comprise much energy, they provide poor acoustic information, while consonant sounds involve little energy but are rich in acoustic cues. Consonant sounds are important as they present high-frequency tone quality and are essential for hearing comprehension.^20^

It should also be pointed out that even subjects with normal hearing as measured by pure tone and speech in quiet tests may have impaired speech recognition in situations of adverse signal-to-noise (S/N) ratios.^21^, ^22^

Tests of sentence recognition in quiet and in noise allow more direct measurements for subjects’ communication abilities,^23^, ^24^, ^25^ thus representing valuable audiological evaluation tools for the analysis of hearing abilities in situations similar to daily auditory experiences.^26^, ^27^, ^28^

This study aimed to evaluate speech recognition in subjects with type I DM in quiet and in competitive noise.

## Methods

The study was approved by the Ethics Committee of the involved institution (protocol number 12609/2012). The application of the audiological examinations lasted, on average, 1 h and 30 min and was concluded on a single day.

### Patients

This was a descriptive, observational and cross-section study conducted with 40 individuals of both genders aged 18–30 years. The participants were divided into two groups: control group (CG), 20 healthy young adults with no hearing complaints or changes, and no systemic diseases; and study group (SG), 20 patients with a diagnosis of type I DM, matched to the control group for age and gender.

The CG was recruited through posters hanging on the walls of the Medical School and through invitations made to the persons accompanying the patients, while the SG subjects were recruited at the Endocrinology Clinic of the involved institution.

The exclusion criteria for both groups were: history of continued exposure to high levels of sound pressure, history of repeated and/or chronic otitis media, otologic surgery, temporal bone trauma, prolonged use of ototoxic drugs, conductive, mixed or sensorineural hearing loss of mild to profound degree.

### Material and procedures

All participants were first submitted to clinical interview and inspection of the ear canal, followed by basic audiological evaluation consisting of pure tone audiometry (PTA) (250–8000 Hz), speech audiometry (Speech Reception Threshold – SRT and the Speech Recognition Index – SRI) and acoustic immittance measures. Speech audiometry was carried out with the subjects wearing headphones.^29^ Immittance audiometry was performed to exclude any middle ear pathology.

All procedures were carried out in an audiometric booth using a two-channel digital audiometer model AC40 (Interacoustics), headphones (TDH39P, Telephonics^®^), and an amplification system for free field audiometry, 110/220 Volts AC current, 50–60 Hz, box power of 100 Watts each. The acoustic immittance measures were carried out by Otoflex 100 (GN Resound) with 3A Insert Earphone.

After the basic audiological evaluation, we applied the List of Sentences in Portuguese test (LSP). This test consists of a list of 25 sentences in Brazilian Portuguese (List 1A), seven lists of 10 sentences each (1B–7B), and speech-spectrum noise.^28^ The sentences and the noise were recorded in independent channels on a compact disk and were played in a Digital Panasonic CD Player, model SL-SX430 coupled to the audiometer. A digital sound pressure level meter instrument, Instrutherm model DEC-420, was used to determine the sound pressure level (SPL) of the sentences and free field noise. To establish the parameters for calibration of the phrase channel, we used the pure tone of the first CD track, in channel one as reference. As for the noise reference, as it is a continuous sound, the noise itself was used for calibration. The output of each channel was calibrated using the VU-meter of the audiometer, with both the pure tone (channel 1) and the noise (channel 2) set at zero level.

The LSP test was applied in free field in an audiometric booth with the participant positioned at a 1-m distance from the sound source at 0° azimuth, with no dislocation on the horizontal or vertical plane. The subject was instructed to repeat each sentence exactly as he or she understood immediately after the presentation. First, the subject was trained to become familiar with the test and its dynamics. The data collected in this training phase was not considered in the analysis of the results.

In the test phase, the response was considered correct when the subject was able to repeat the presented sentence in full, with no errors or omissions. Thus, when the answer was correct, the intensity of the sentence was reduced by 4 dB. The use of 4 dB intervals is recommended for the presentation of the stimuli until the first change in the type of response occurs, and then 2 dB intervals until the end of the list. This procedure was followed for the measurements in quiet (List 1B) and in noise (List 2B), and the presentation levels of each sentence were recorded. The final result of the test is represented by the mean value of the intensity levels of the sentence presentation, calculated from the degree of performance that occurred at the first change in the type of response to the level of presentation of the last sentence on the list. The signal to noise ratio (S/N ratio) was obtained by subtracting the standard of the intensity of noise present (fixed at 65 dBHL) from the mean intensity of sentence presentation. Thus, the S/N ratio corresponded to the difference in dB between the speech recognition threshold test in noise (SRTN) value and the competitive noise value. The S/N ratio was identified as the level at which the subject was able to recognize about 50% of the sentences presented. Different lists used with the intention of eliminating the possibility of a better performance due to sentence memorization.^28^

### Statistical analysis

The mean audiometric thresholds were analyzed according to the band frequencies, i.e. L1 (250 Hz to 1 kHz), L2 (2–4 kHz) and L3 (6–8 kHz). Analysis of variance (ANOVA) with repeated measures was used to compare the audiometric thresholds, the speech reception thresholds and the speech recognition index between the two groups.

The Student's *t*-test for paired quantitative data used for intragroup comparison of mean Speech Recognition Threshold Test in Quiet (SRTQ), and SRTN values, and the Student's *t*-test for independent data used for intergroup comparison of mean SRTQ and SRTN values. The level of significance set at 5% for all analyses.

## Results

A total of 40 participants, aged 18–30 years (23 years average) were evaluated: 20 type 1 diabetics and 20 healthy control group, matched for age and gender. The duration of the disease was <5 years in 15%, 5–15 years in 55% and >15 years in 30% the patients, respectively.

All participants had normal bilateral audiometric thresholds.^29^ When the audiometric thresholds, the speech reception thresholds and the speech recognition index were compared between the right and left ears of the two groups, there was no evidence of a statistically significant difference (ANOVA), which allowed pooling the results for each ear into a single sample.

There was no significant difference in audiometric thresholds according to band frequency between the two groups (250 Hz to 1 kHz, *p* = 0.12; 2–4 kHz, *p* = 0.79; and 6–8 kHz, *p* = 0.89).

The speech audiometry tests were compatible with pure tone threshold, an expected result since both groups had normal hearing acuity.^29^

All participants had a type A tympanometry curve indicating normal mobility of the tympanic-ossicular system.^30^

Mean SRTQ, SRTN and S/N ratio were 25.79, 49.03 and −15.96 dBHL for CG subjects and 35.69, 62.62 and −2.38 dBHL for SG subjects, respectively.

**Figure 1:**
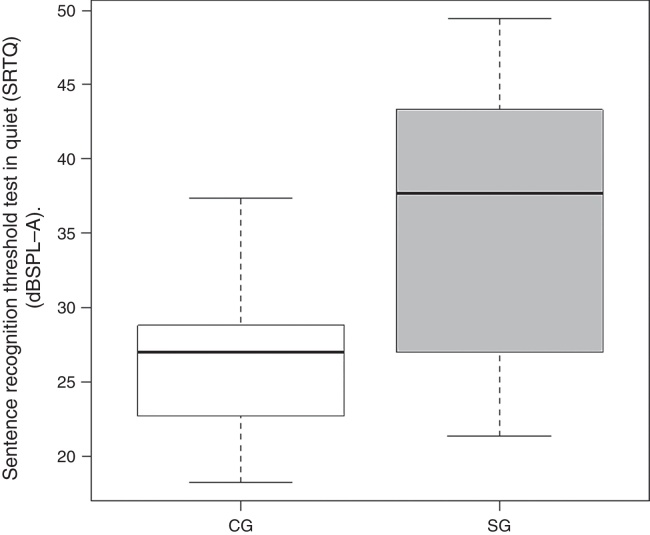
Comparison of SRTQ results between CG and SG. Note: Control group (CG), study group (SG).

**Figure 2:**
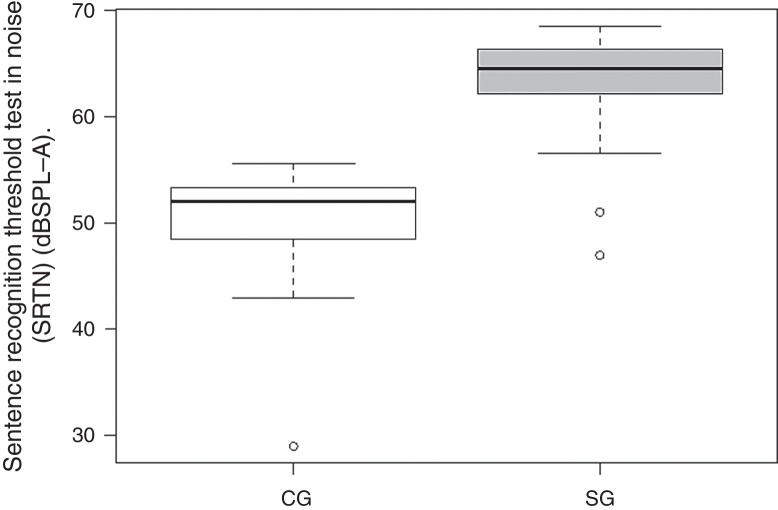
Comparison of mean SRTN between CG and SG. Note: Control group (CG), study group (SG).

Comparison of mean SRTQ and SRTN values revealed a significant difference between CG and SG subjects (Figure 1, Figure 2).

**Figure 3:**
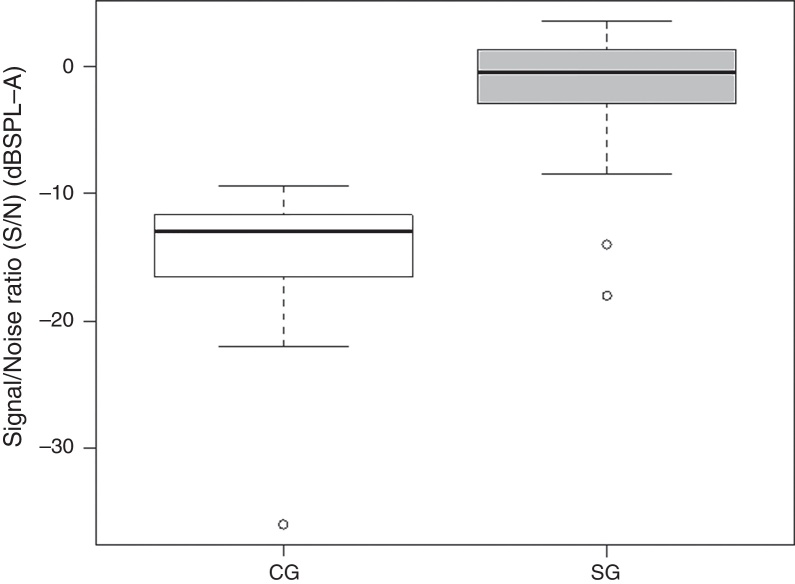
Comparison of S/N ratios between CG and SG. Note: Control group (CG), study group (SG).

Comparison of the S/N ratio revealed a significant difference (*p* < 0.0001) between CG and SG subjects (Fig. 3).

Intragroup comparison of mean SRTQ and SRTN through Student *t*-test revealed a significant difference (*p* < 0.0001) for all participants (*n* = 80 ears).

## Discussion

Currently, there is no consensus in the specialized literature from the audiological and histopathological viewpoint concerning the correlation of DM with hearing loss, i.e. there is no sufficient scientific evidence characterizing a clear cause–effect relationship.

Several studies have investigated whether there is hearing loss inherent to DM and which factors can be associated with this loss. The studies are controversial and highly variable regarding the incidence of hearing loss in patients with diabetes.^5^

In the present study, there was no difference in mean tone hearing thresholds between diabetic subjects and control group subjects.

In a study on the influence of DMI on the hearing of young adults, Doricci^6^ applied pure tone audiometry threshold to healthy and diabetic subjects and found a statistically significant increase in mean pure tone thresholds at all frequencies studied in diabetic patients.

Marchiori and Gibrin^31^ identified a larger number of diabetic subjects with hearing loss compared with healthy subjects. However, their sample consisted of patients ranging from 33 to 84 years, in contrast to the present example, which consisted of young subjects aged on average 23 years.

According to Maia and Campos,^5^ the decline of hearing acuity in elderly diabetic individuals is even higher than would be expected for their age due to presbyacusis.

When young subjects with DMI, with short duration of the disease and without evident clinical hearing manifestations were evaluated, their pure tone thresholds were found to be significantly higher at high frequencies and partially higher at medium frequencies compared to healthy subjects.^3^ Similar findings were obtained in other studies.^1^, ^2^, ^3^, ^5^, ^6^

Henriques and Costa^32^ warned that obtaining pure tone threshold and applying tests with separate words is not sufficient for a more extensive and reliable detection of an individual's communicative ability. Therefore, applying sentence recognition tests in quiet and in noise is essential as they permit the analysis of the hearing abilities in situations similar to real daily hearing experiences.

We found a statistically significant difference in mean SRTQ, SRTN and S/N ratio between the subjects with and without DMI. In the literature review, we did not find any studies investigating speech perception in quiet and in noise in diabetic subjects using the LSP test.^20^ The speech perception tests applied to DMI subjects consisted of monosyllabic words in quiet and in noise. The speech perception ability of these subjects reduced for words in quiet and particularly in the presence of noise compared to control, with 20% lower scores.^33^ These results confirm the importance of speech perception analysis in competitive noise, and illustrate that the use of sentences is more appropriate than simply words, because the use of sentences resembles the participants’ daily listening experience.

The temporal resolution hearing abilities of subjects with DMI may be impaired and may thus explain how these subjects are less able to use relatively rapid periods of quiet within the fluctuating environmental noise in order to understand the speech signal.^33^, ^34^ The statistically significant difference observed in the sentence recognition tests in quiet and in noise applied to the participants with and without DM I in the absence of hearing complaints, hearing loss or both, reveals that impairment of speech perception in both contexts (quiet and noise) may occur due to changes in the functioning of the central auditory system as a consequence of DM. Further studies are necessary to investigate speech perception with a competitive message in diabetic subjects.

The difference in sentence recognition between the presence and absence of noise was also observed in the control group. Young people with typical hearing and no clinical complaints of difficulty in understanding speech may show impaired speech recognition in situations of adverse S/N ratios. This problem may be attributed, in part, to the negative effects of noise on neural synchrony, which result in degraded speech representation at cortical and subcortical levels.^35^

Various hearing abilities are required to reach the same degree of speech recognition, demonstrating that more detailed sensory information is necessary under difficult listening conditions.^36^ Further studies are needed to elucidate the presence of impairment in the peripheral and central auditory system, especially regarding possible damage to speech recognition for different listening conditions in diabetic subjects. Audiologic follow up of subjects with DM is recommended.

## Conclusion

Speech recognition performance in quiet and in competitive noise was worse in subjects with type I diabetes mellitus compared to subjects without diabetes.

## Conflicts of interest

The authors declare no conflicts of interest.
